# Factors associated with the development of recurrent and contralateral Charcot neuroarthropathy in individuals with diabetes mellitus: A scoping review

**DOI:** 10.1002/jfa2.70016

**Published:** 2024-11-17

**Authors:** Keet Yeng Cheong, Shan M. Bergin, Shannon E. Munteanu, Byron M. Perrin, Karl B. Landorf

**Affiliations:** ^1^ Discipline of Podiatry School of Allied Health Human Services and Sport La Trobe University Melbourne Victoria Australia; ^2^ La Trobe Rural Health School La Trobe University Bendigo Victoria Australia

**Keywords:** ankle, charcot joint, diabetes mellitus, foot, recurrence

## Abstract

**Introduction:**

Charcot neuroarthropathy (CN) can result in fractures and dislocations of the foot and ankle in individuals with diabetes and neuropathy, leading to ulceration, amputation and a poor quality of life. Additional episodes of acute CN can lead to extended periods of physical and psychosocial distress. The aim of this scoping review was to identify and synthesise the evidence relating to factors associated with the development of recurrent and contralateral Charcot neuroarthropathy (CN) in individuals with diabetes.

**Methods:**

A systematic search of four electronic databases was conducted from inception to February 06, 2023. All relevant study designs, except single case studies, that had been published in full in peer‐reviewed journals were included. Studies were excluded if they were not published in English and did not provide data on individuals with diabetes.

**Results:**

The search identified two studies that investigated factors associated with the development of recurrent CN, but none that related to the development of contralateral CN. Ten factors were investigated for association with recurrent CN development: age, body mass index, diabetes type and duration, glycated haemoglobin, anatomical site affected, duration of offloading applied to treat the primary CN episode, use of pharmacological intervention, severity of neuropathy, and skin temperature. However, no significant associations were reported.

**Conclusions:**

There is an alarming lack of evidence‐based findings in this research area to guide practice. Clearly, more research in the form of rigorous prospective studies is urgently required to identify risk factors for the development of recurrent and contralateral CN in individuals with diabetes.

## 1 INTRODUCTION

Charcot neuroarthropathy (CN) is a destructive and debilitating condition that affects bones, joints and soft tissues [1]. In the acute phase of the condition, it is characterised by uncontrolled inflammation and osteopenia that can give rise to localised fractures and joint dislocation [2]. The foot and ankle is a common region for CN to occur where it can progress to severe permanent deformity such as collapse of the midfoot and malformation of the ankle.

Peripheral neuropathy is thought to be the key initiating factor for CN and is associated with more than a dozen medical conditions such as alcoholism, Charcot–Marie–Tooth disease and leprosy [3]; however, diabetes mellitus is the most common underlying cause of neuropathy [4, 5]. CN predominantly affects the foot and ankle of individuals with diabetes [2, 6], with prevalence estimates reported to range from 0.04% of patients attending foot care specialist centres in England [7], to 0.3% of patients attending a regional referral centre in Ireland [8], to 0.56% of all individuals with diabetes in a national registry in Denmark [9]. The long‐term impact of CN on affected individuals is substantial, with high rates of foot ulceration and amputation [10, 11], and poor quality of life [11, 12].

Once an individual experiences an episode of CN, further episodes of acute CN can occur in two ways including (i) *recurrence* of the condition in the ipsilateral (same) foot and/or ankle or (ii) occurrence of the condition in the *contralateral* (opposite) foot and/or ankle [13]. Further episodes of acute CN, whether recurrent or contralateral episodes, can cause considerable burden, with studies reporting extended duration of treatment and poor outcomes in those affected including high rates of foot ulceration [14, 15, 16]. Consequently, the burden associated with recurrent and contralateral CN may contribute further to the enormous economic costs associated with diabetes‐related foot disease [17]. Despite this, little research has investigated the factors associated with the development (or causes) of further episodes of CN in individuals with diabetes. For example, associated factors such as patient characteristics/comorbidities and the management of the initial episode of CN may provide valuable information. Identifying and subsequently managing these factors may lead to more effective treatment or even the prevention of recurrent or contralateral CN, thereby decreasing the overall burden of CN.

To the authors' knowledge, no scoping reviews have investigated the factors associated with the development of recurrent or contralateral CN in individuals with diabetes. Therefore, the aim of this study was to identify and synthesise the evidence relating to factors associated with the development of recurrent and contralateral CN in the foot or ankle of individuals with diabetes.

## 2 METHODS

### 2.1 Protocol

This study was a scoping review, and its protocol was developed using the Joanna Briggs Institute (JBI) Manual for Evidence Synthesis [18]. The scoping review was reported with reference to the Preferred Reporting Items for Systematic Reviews and Meta‐analyses extension for scoping reviews (PRISMA‐ScR) guidelines [19].

### 2.2 Eligibility criteria

To be included in this scoping review, studies must have been published in full in a peer‐reviewed journal. Included studies must have reported or investigated factors associated with the development of recurrent or contralateral CN in the foot or ankle of individuals with diabetes. All relevant study designs including randomised controlled trials, cohort studies, case‐control studies, cross‐sectional studies and case series were eligible for inclusion. Single case studies were excluded as they are the lowest form of evidence and would have provided poor information relative to the aims of the study.

Studies were also excluded if they (i) included individuals with CN that was not attributable to diabetes, unless data from individuals with diabetes was isolated and analysed and (ii) were not published in English language.

### 2.3 Information sources and search strategy

A systematic search of the literature was performed by one of the authors (KYC) from inception to February 06, 2023 using MEDLINE, EMBASE, Cumulative Index to Nursing and Allied Health Literature (CINAHL) and Allied and Complementary Medicine Database (AMED) databases. Four core concepts comprising of Medical Subject Headings (MeSH) terms, keywords and synonyms were utilised in the search strategy: (i) “diabetes mellitus” AND (ii) “neurogenic arthropathy” AND (iii) “foot OR ankle” AND (iv) “recurrence or contralateral”. Boolean operators and wildcards were applied to expand the search. The search strategy used for all electronic databases is attached in Supplementary File 1.

### 2.4 Selection of sources of evidence and data extraction

The final search results were exported to Covidence [20], and duplicates were removed. Title and abstract screening were performed by two authors independently (KYC and BMP), and full text copies of all relevant articles were obtained for further assessment. Disagreements were resolved by discussion and consensus with a third author (KBL). The reference lists of all included studies were also hand‐searched for eligible studies to supplement the electronic database search.

### 2.5 Data charting process and data items

The following data were extracted from all included studies: study characteristics (e.g. design, aim, duration and location), participant characteristics (e.g. age, sex, body mass index [BMI], CN and diabetes characteristics), methods reported for confirming diagnosis and resolution of acute CN, treatment provided for CN, associated factor(s) investigated, and statistical analyses used. Descriptive statistics and inferential statistics were extracted if available. Data extraction was performed by KYC and recorded within a Microsoft^®^ Excel^®^ spreadsheet (Microsoft Corporation, Redmond, Washington, United States) using a standardised template. To ensure that the data were accurately extracted, data verification was performed independently by two authors (SMB and SEM), and any discrepancies were discussed.

### 2.6 Synthesis of results

Due to this being a scoping review, no pooling of data was attempted, and a narrative synthesis of the study findings was performed. Moreover, data could not be pooled from included studies due to the heterogeneous nature of the study designs and variables of interest. Studies were grouped by the outcome of interest (i.e. recurrent CN or contralateral CN) and the type of associated factor they investigated. The associated factors were categorised as follows: (i) initial treatment of acute CN, (ii) participant characteristics and comorbidities and (iii) CN characteristics and others.

## 3 RESULTS

### 3.1 Selection of studies

**FIGURE 1:**
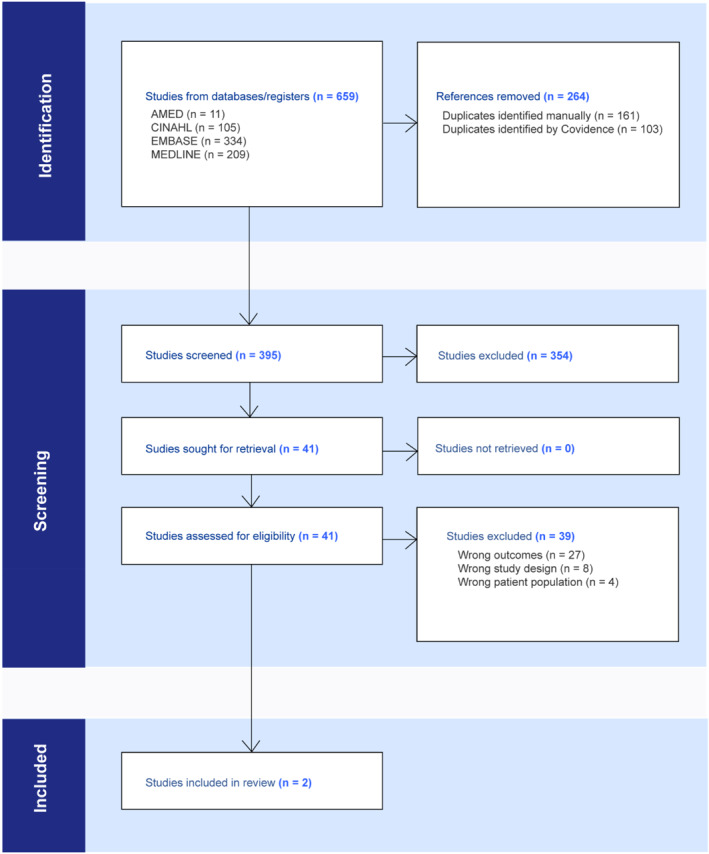
PRISMA flow chart.

A total of 659 studies were identified from the search conducted. The process of article screening is shown in the Preferred Reporting Items for Systematic reviews and Meta‐Analyses (PRISMA) flow chart [21] in Figure 1. Following the removal of 264 duplicate references, title and abstract screening was performed for 395 studies. Of these, 354 studies were excluded. Full text screening was performed for the remaining 41 studies, and 39 studies were excluded, resulting in 2 studies being included for full text extraction. No additional studies were identified from handsearching the reference lists of these studies.

### 3.2 Characteristics of studies

This review identified two studies (by Christensen et al. [14] and Busch‐Westbroek et al. [22]) that investigated factors associated with recurrent CN development and no studies that investigated factors associated with contralateral CN development. The study designs included a single‐centre observational cross‐sectional study [14], and a single‐centre observational study with historic controls [22]. Both studies were conducted in hospitals, but in different countries, Denmark [14] and the Netherlands [22]. Study duration was similar for both studies (5 years for Christensen et al. [14] and 4 years for Busch‐Westbroek et al. [22]), and the unit of allocation was individual participants. Inclusion criteria were reported in both studies [14, 22], but exclusion criteria were not reported.

### 3.3 Participant characteristics

Information relating to participant characteristics measured at baseline is reported in Table 1. Both studies [14, 22] included participants with CN and diabetes. The total number of analysed participants with diabetes was 56 participants [14] and 22 participants [22]. In the study by Christensen et al. [14], there were a total of 33 male and 23 female participants, with a mean age of 56.2 ± 14.1 years and 58.8 ± 11.0 years for participants with and without CN recurrence, respectively. In the study by Busch‐Westbroek et al. [22], there were a total of 10 male and 12 female participants, with a mean age of 59 ± 7 years and 56 ± 16 years for participants in the intervention group and the usual care group, respectively. Mean BMI in the study by Christensen et al. [14] was 29.3 ± 5.8 kg/m^2^ and 28.2 ± 5.1 kg/m^2^ for participants with and without CN recurrence, respectively, and in the study by Busch‐Westbroek et al. [22], it was 33 ± 5 kg/m^2^ and 26 ± 6 kg/m^2^ for participants in the intervention group and the usual care group, respectively. The study follow‐up period was a mean of 3.2 years by Christensen et al. [14] and 1 year by Busch‐Westbroek et al. [22]. Participant comorbidities and biochemistry analysis were reported in the study by Busch‐Westbroek et al. [22] but not in the study by Christensen et al. [14]. Both studies [14, 22] reported on diabetes type, mean duration of diabetes and mean glycated haemoglobin (HbA_1c_).

**TABLE 1:** Baseline characteristics of participants in studies that investigated factors associated with recurrent CN development.

Authors (date)	Analysed participants with CN and follow‐up duration	Group	Analysed participants by diabetes type	Age, mean ± SD, years	Sex	BMI, mean ± SD, kg/m^2^	HbA_1c_, mean ± SD, %	Diabetes duration, mean ± SD, years	Comorbidities	Biochemistry
Christensen et al. (2012)	*n* = 56 (100%) Mean duration = 3.2 years (no SD reported)	Overall sample	Type 1 diabetes, *n* = 24 (42.9%) Type 2 diabetes, *n* = 32 (57.1%)	58.3 ± 11.6	Male, *n* = 33 (58.9%) Female, *n* = 23 (41.1%)	28.4 ± 5.2	8.9 ± 1.7	Overall: 24.5 ± 13.6 Type 1 diabetes: 34.4 ± 13.5 Type 2 diabetes: 17.1 ± 7.8	Not reported	Not reported
		CN recurrence group	Type 1 diabetes, *n* = 6 (25.0%) Type 2 diabetes, *n* = 4 (12.5%)	56.2 ± 14.1	Not reported	29.3 ± 5.8	9.3 ± 1.4	Overall: 25.1 ± 11.1 Type 1 diabetes: 27.7 ± 14.1 Type 2 diabetes: 21.3 ± 2.8	Not reported	Not reported
		No CN recurrence group	Type 1 diabetes, *n* = 18 (75.0%) Type 2 diabetes, *n* = 28 (87.5%)	58.8 ± 11.0	Not reported	28.2 ± 5.1	8.7 ± 1.7	Overall: 24.4 ± 14.2 Type 1 diabetes: 36.7 ± 12.8 Type 2 diabetes: 16.5 ± 8.1	Not reported	Not reported
Busch‐Westbroek et al. (2018)	*n* = 22 (100%) Duration = 1.0 years (predetermined follow‐up)	Overall sample	Type 1 diabetes, *n* = 6 (27.3%) Type 2 diabetes, *n* = 16 (72.7%)	Not reported	Overall sample Male, *n* = 10 (45.5%) Female, *n* = 12 (54.5%)	Not reported	Not reported	Not reported	Neuropathy *n* = 22 Retinopathy *n* = 17 Albuminuria *n* = 14 Peripheral arterial occlusive disease *n* = 2 Alendronate (Bisphosphonate) use *n* = 5	Not reported
		Denosumab group	Type 1 diabetes, *n* = 1 (16.7%) Type 2 diabetes, *n* = 10 (62.5%) The number of participants with type 2 diabetes was significantly higher in the Denosumab group (*p* < 0.05)	59 ± 7	Male, *n* = 7 (70.0%) Female, *n* = 4 (33.3%)	33 ± 5	8.1 ± 1.4	16 ± 12	Neuropathy *n* = 11 (100%) Retinopathy *n* = 8 (72.7%) Albuminuria *n* = 7 (63.6%) Peripheral arterial occlusive disease *n* = 1 (9.1%) Alendronate (Bisphosphonate) use *n* = 0	Creatinine clearance (ml/min) 117 ± 60 Calcium (mmol/L) 2.5 ± 0.1 Albumin (g/L) 45 ± 3 25‐OH vitamin D (nmol/L) 51 ± 13
		Usual care group	Type 1 diabetes, *n* = 5 (83.3%) Type 2 diabetes, *n* = 6 (37.5%)	56 ± 16	Male, *n* = 3 (30.0%) Female, *n* = 8 (66.7%)	26 ± 6 BMI was significantly higher in the Denosumab group. (*p* < 0.05)	8.2 ± 1.2	21 ± 8	Neuropathy *n* = 11 (100%) Retinopathy *n* = 9 (81.8%) Albuminuria *n* = 7 (63.6%) Peripheral arterial occlusive disease *n* = 1 (9.1%) Alendronate (Bisphosphonate ^a^ ) use *n* = 5 (45.5%)	Creatinine clearance (ml/min) 91 ± 27 Calcium (mmol/L) 2.4 ± 0.1 Albumin (g/L) 44 ± 3 25‐OH vitamin D (nmol/L) 38 ± 17

*Note:* Abbreviations: BMI, body mass index; CN, Charcot neuroarthropathy; HbA_1c_, glycated haemoglobin; SD, standard deviation.

^a^ Bisphosphonates are sometimes used in CN to potentially inhibit bone resportion [1].

### 3.4 Diagnosis and management of CN

Information about CN diagnosis and treatment for the two studies is summarised in Table 2. Christensen et al. [14] reported on the methods used for diagnosis of the primary episode of CN, including clinical assessment, radiographs, bone scintigram and a positive response to offloading, and confirmation of the resolution of the acute phase via clinical assessment. In this study [14], the method used for diagnosis of the recurrent CN event was also reported and involved the use of clinical assessments to detect new foot swelling and skin temperature difference greater than 2°C at least 1 month after full weightbearing. Diagnostic information was not reported by Busch‐Westbroek et al. [22].

**TABLE 2:** Details of CN diagnosis and treatment in studies that investigated factors associated with recurrent CN development.

Authors (date)	Diagnosis of primary CN episode	Resolution of primary acute CN episode	Treatment received	Definition of acute CN recurrence	Diagnosis of acute CN recurrence
Christensen et al. (2012)	Clinical assessment X‐rays Bone scintigram Positive response to offloading	Temperature difference <2°C at two follow‐up visits, oedema subsided	Offloading with crutches and removable walkers with custom orthotics Indoor ambulation permitted Could remove walkers at night unless ankle affected or gross instability present Treatment response monitored by reviewing skin temperature and degree of oedema every 2–6 weeks Gradual return to the weightbearing post‐acute phase supported by sandals with rigid roller bars, custom insole, footwear and/or brace CN recurrence was treated like the primary episode with crutches and removable walker	New swelling and skin temperature difference >2°C in the same foot after at least 1 month of full weightbearing	Clinical assessments New foot swelling Skin temperature difference >2°C at least 1 month after full weightbearing
Busch‐Westbroek et al. (2018)	Not reported	Not reported	Weekly total contact casting X‐rays every 4 weeks Calcium 500 mg/cholecalciferol 800 IE daily Treatment group received single injection of Denosumab	Not reported	Not reported

*Note:* Abbreviation: CN, Charcot neuroarthropathy; IE, internationale eenheid (the same as international units).

Clinical management for the primary episode of acute CN was described in both studies [14, 22]. Busch‐Westbroek et al. [22] applied a total contact cast to all participants and administered an injection of anti‐resorptive drug (Denosumab) to increase bone strength [23] to the intervention group (referred hereon as the Denosumab group). Christensen et al. [14] applied a removable cast walker and custom foot orthosis to all participants (Table 2). In this study [14], participants were allowed to ambulate indoors with crutches while wearing the offloading devices and were encouraged to undertake physical training on a bicycle or an exercise bike while wearing them. Christensen et al. [14] also described the management plan following resolution of the primary episode of acute CN and for the recurrent CN episode (Table 2).

### 3.5 CN characteristics

A summary of the CN characteristics of participants in the two studies [14, 22] is provided in Table 3. In terms of the *clinical stage*, both studies [14, 22] only included participants with CN in the acute phase at the time of clinical presentation. The *anatomical site* affected by the primary episode of CN was reported by both studies [14, 22], with Busch‐Westbroek et al. [22] applying the modified Brodsky classification system [24], which comprises of six classifications that relate to anatomical sites (Table 3). The most common site recorded was the midfoot in both studies [14, 22], also classified in the study by Busch‐Westbroek et al. [22] as Type 1: Midfoot (tarsometatarsal and naviculocuneiform joints) in the modified Brodsky classification. The rate of CN recurrence among participants with diabetes in the two studies was 9% [22] and 18% [14], respectively. The *anatomical site* of the recurrent episode was reported by Christensen et al. [14]. Similar to the primary episode of CN, the most common anatomical site recorded for recurrent CN was the midfoot [14].

**TABLE 3:** CN characteristics of participants in studies that investigated factors associated with recurrent CN development.

Authors (date)	Primary CN episode			Recurrent CN episode		
	Analysed participants	Clinical stage, classification system used	Anatomical classification, classification system used	Analysed participants	Clinical stage (classification system used and numbers)	Anatomical classification (classification system used and numbers)
Christensen et al. (2012)	CN recurrence group *n* = 10 (17.9%) No CN recurrence group *n* = 46 (82.1%)	No classification system described All in acute clinical stage, *n* = 56 (100%)	No classification system described Overall sample: Forefoot: *n* = 15 (26.8%) Midfoot: *n* = 31 (55.4%) Heel: *n* = 3 (5.4%) Ankle: *n* = 7 (12.5%) Not reported for groups with and without CN recurrence	*n* = 10 (17.9%)	Not reported	No classification system described Forefoot: *n* = 2 (20.0%) Midfoot: *n* = 7 (70.0%) Ankle: *n* = 1 (10.0%)
Busch‐Westbroek et al. (2018)	Denosumab group *n* = 11 (50.0%) Usual care group *n* = 11 (50.0%)	No classification system described All in acute clinical stage, *n* = 22 (100%)	Modified Brodsky classification^a^ Denosumab group Type 1, *n* = 8 (72.7%) Type 2, *n* = 0 Type 3A, *n* = 0 Type 3B, *n* = 0 Type 4, *n* = 2 (18.2%) Type 5, *n* = 1 (9.1%) Usual care group Type 1, *n* = 6 (54.5%) Type 2, *n* = 0 Type 3A, *n* = 0 Type 3B, *n* = 0 Type 4, *n* = 3 (27.3%) Type 5, *n* = 2 (18.2%)	Denosumab group, *n* = 0 Usual care group, *n* = 2 (9.1%)	Not reported	Not reported

*Note:* Abbreviation: CN, Charcot neuroarthropathy.

^a^ Modified Brodsky classification: Type 1: midfoot (tarsometatarsal and naviculocuneiform joints); Type 2: hindfoot (talonavicular, calcaneocuboid and/or subtalar joints); Type 3A: ankle (tibiotalar joint); Type 3B: calcaneus (tuberosity fracture of the calcaneus); Type 4: multiple regions (sequential and concurrent); Type 5: forefoot (metatarsophalangeal joints) [28].

### 3.6 Factors investigated for recurrent CN development

#### 3.6.1 Initial treatment of acute CN

Both studies [14, 22] in this review investigated initial treatments used for acute CN, such as offloading and monoclonal antibodies, to evaluate the role they might play in the development of recurrent CN. Christensen et al. [14] investigated the duration of offloading for the primary acute CN episode and found no statistically significant difference between the group with CN recurrence and the group with no CN recurrence (142 ± 24 days vs. 134 ± 41 days). Busch‐Westbroek et al. [22] assessed the effect of Denosumab for increasing bone strength [23] on recurrent CN development and found that the Denosumab group had a reduced rate of CN recurrence. However, this difference was not found to be statistically significant [22].

#### 3.6.2 Participant characteristics and comorbidities

Christensen et al. [14] investigated age, BMI, type and duration of diabetes, and HbA_1c_ as associated factors with recurrent CN development, but no statistically significant differences were reported between the groups with and without CN recurrence (Table 1). Busch‐Westbroek et al. [22] did not investigate patient characteristics and comorbidities.

#### 3.6.3 CN characteristics and other factors

Christensen et al. [14] also investigated associations with the anatomical site of the primary CN episode, temperature difference between the anatomical site with the highest skin temperature and its corresponding contralateral site, and severity of neuropathy as measured by biothesiometry for recurrent CN development. Again, no statistically significant differences between the groups with and without CN recurrence were reported [14].

### 3.7 Synthesis of results

All factors that were investigated for association with recurrent CN development in participants with diabetes are summarised in a table in Supplementary File 2. A total of 10 factors (outlined above and in Supplemntary File 2) were investigated, but none were found to be significantly associated with recurrent CN. The results of the statistical tests of association reported by Christensen et al. [14] and Busch‐Westbroek et al. [22] can be found in Supplementary File 3.

## 4 DISCUSSION

### 4.1 Summary of evidence

The aim of this scoping review was to identify and synthesise the evidence relating to factors associated with the development of recurrent and contralateral CN in the foot or ankle of individuals with diabetes. The review identified only two studies [14, 22] that investigated factors associated with recurrent CN development, and did not find any eligible studies that investigated factors associated with contralateral CN development.

The factors investigated for association with recurrent CN development included (i) age, (ii) BMI, (iii) diabetes type, (iv) diabetes duration, (v) HbA_1c_, (vi) anatomical site of the primary CN episode, (vii) temperature difference between the anatomical site with the highest skin temperature and its corresponding contralateral site, (viii) severity of neuropathy measured by biothesiometry, (ix) duration of offloading for the primary episode of acute CN [14] and (x) pharmacological treatment with Denosumab [22]. However, none of these factors were found to be significantly associated with rates of recurrent acute CN in individuals with diabetes. This may be attributed to the small sample sizes and relatively short follow‐up durations of the two studies [14, 22], which may have resulted in a lower frequency of recurrent CN cases being detected.

The rate of CN recurrence among participants with diabetes in the two studies was 9% after a follow‐up of 1 year [22] and 18% after a mean follow‐up of 3 years [14]. These recurrence rates are comparable to other studies; Fabrin et al. [13] reported a 17% rate after a median follow‐up of 4 years, and Osterhoff et al. [25] reported a 24% rate after a mean follow‐up of 4 years. However, it is possible that the recurrence rate of acute CN may be underestimated as the acute phase of the disorder is often misdiagnosed [1, 26]. The development of recurrent CN in up to one in four individuals within 4 years in these studies [13, 14, 22, 25] emphasises the importance of being aware of the disorder, and ensuring that long‐term foot surveillance is instituted for timely recognition and management of acute CN.

The results of this review highlight that there is a critical lack of research relating to factors associated with the development of recurrent and contralateral CN in individuals with diabetes. When interpreted in the context of the extended treatment times, poor patient outcomes (including high rates of foot ulceration, amputation and mortality) and economic burden that relate to CN [10, 11, 14, 15, 16, 17, 27], this gap in research is particularly concerning. Additional episodes of CN can subject previously affected individuals to prolonged treatment time, which can extend over many months [14, 15, 16]. In addition, affected individuals often undergo extensive treatment, including total contact casting [15, 16], which significantly disrupt activities of daily living [2]. Additional episodes of CN also increase the likelihood of poor outcomes including a high rate of foot ulceration [16].

In a recent study, Gooday et al. [2] found that individuals with diabetes and acute CN experienced substantial physical and psychosocial changes, which were most commonly brought about by the use of a below‐knee offloading device for an extended duration. These changes included social isolation, disruptions to their daily roles, responsibilities, relationships and mobility, and pain associated with the use of the offloading device [2]. Given these findings, it is likely that repeated episodes of CN, whether recurrent or contralateral, contribute further to extended periods of physical and psychosocial distress; however, further research is needed to investigate this.

The paucity of research relating to factors associated with the development of recurrent and contralateral CN means that there is little evidence to guide clinicians managing patients with diabetes and CN. This critical lack of research highlights the challenges associated with identifying patients who may be more susceptible to developing these complications and implementing strategies to mitigate their risk. Given the significant morbidity and mortality associated with CN, there is an urgent need for high‐quality research to address these knowledge gaps.

### 4.2 Strengths and limitations

The strengths of this scoping review include its search of multiple key databases, well developed search strategy, stringent inclusion and exclusion criteria, rigorous article screening process, and screening and data extraction by two independent reviewers. These strengths have enabled the review to highlight the lack of high‐quality evidence relating to factors associated with recurrent and contralateral CN development in individuals with diabetes. However, there are two limitations of the review that also need to be considered. Firstly, the review did not perform a search of grey literature, so it may have missed some studies in the yield. Secondly, the review excluded studies that were not published in English, so studies published in other languages may have been missed.

## 5 CONCLUSIONS

This scoping review identified only two eligible studies that had investigated factors associated with the development of recurrent CN and no studies that investigated factors associated with the development of contralateral CN in individuals with diabetes. Further, none of the associations among the investigated factors were statistically significant. Therefore, there is an alarming lack of research related to recurrent and contralateral CN, and there is minimal evidence to guide clinicians attempting to reduce these complications from occurring. Further high‐quality research is urgently needed to determine risk factors for these disabling and highly burdensome sequelae of CN in individuals with diabetes. This is important because identifying individuals at risk of recurrent or contralateral CN will help with their management, and ultimately may lead to prevention of the conditions.

## AUTHOR CONTRIBUTIONS

**Keet Yeng Cheong:** Conceptualisation; methodology; writing—original draft; data collection; data analysis; writing—review and editing. **Shan M. Bergin:** Conceptualisation; methodology; data collection; data analysis; writing—review and editing. **Shannon E. Munteanu:** Conceptualisation; methodology; data collection; data analysis; writing—review and editing. **Byron M. Perrin:** Data collection; data analysis; writing—review and editing. **Karl B. Landorf:** Conceptualisation; methodology; data collection; data analysis; writing—review and editing.

## FUNDING INFORMATION

None to declare.

## CONFLICT OF INTEREST STATEMENT

KBL is an emeritus editor, SMB is an associate editor, and SEM is a previous deputy editor of the *Journal of Foot and Ankle Research*.

## ETHICS STATEMENT

Not applicable.

## CONSENT FOR PUBLICATION

Not applicable.

## Data Availability

The data that support the findings of this study are available from the corresponding author upon reasonable request.
